# Coronary artery calcium in patients with schizophrenia

**DOI:** 10.1186/s12888-021-03412-x

**Published:** 2021-08-23

**Authors:** Trine Trab, Rubina Attar, Svend Eggert Jensen, Simon Grøntved, Jens Brøndum Frøkjær, Christoffer Polcwiartek, René Ernst Nielsen

**Affiliations:** 1 Department of Psychiatry, Aalborg University Hospital, Aalborg, Denmark; 2 Department of Cardiology, Aalborg University Hospital, Aalborg, Denmark; 3 Department of Clinical Medicine, Aalborg University, Aalborg, Denmark; 4 Department of Radiology, Aalborg University Hospital, Aalborg, Denmark

**Keywords:** Coronary artery disease, Schizophrenia, Vascular Calcification

## Abstract

**Background:**

Coronary heart disease (CHD) is a major cause of increased mortality rates in patients with schizophrenia. Moreover, coronary artery calcium (CAC) score is associated with CHD. We hypothesized that patients with schizophrenia have more CAC than the general population and aimed to investigate the CAC score in patients with schizophrenia compared to norms based on the general population. Additionally, this study investigated if age, sex, diabetes, dyslipidemia and smoking were associated with the CAC score.

**Methods:**

In a cross-sectional study, 163 patients with schizophrenia underwent cardiac computed tomography, and the CAC score was measured and compared to norms by classifying the CAC scores in relation to the age- and gender matched norm 50th, 75th and 90th percentiles. Logistic and linear regression were carried out to investigate explanatory variables for the presence and extent of CAC, respectively.

**Results:**

A total of 127 (77.9%) patients had a CAC score below or equal to the matched 50th, 20 (12.3%) above the 75th and nine (5.5%) above the 90th percentile. Male sex (*P* < 0.05), age (*P* < 0.001) and smoking (*P* < 0.05) were associated with the presence of CAC while age (*P* < 0.001) and diabetes (*P* < 0.01) were associated with the extent of CAC.

**Conclusions:**

The amount of CAC in patients with schizophrenia follows norm percentiles, and variables associated with the CAC score are similar in patients with schizophrenia and the general population. These findings indicate that the CAC score may not be sufficient to detect the risk of CHD in patients with schizophrenia. Future studies should explore other measures of subclinical CHD, including measures of peripheral atherosclerosis or cardiac autonomic neuropathy to improve early detection and intervention.

**Trial registration:**

ClinicalTrials.gov Identifier: NCT02885792, September 1, 2016.

**Supplementary Information:**

The online version contains supplementary material available at 10.1186/s12888-021-03412-x.

## Background

Compared to the general population, patients with schizophrenia have a shorter life expectancy and experience relatively more deaths from unnatural causes, e.g. suicide [1, 2]. However, most life years lost are attributable to physical disease including coronary heart disease (CHD) [1–3]. Patients with schizophrenia have an increased prevalence of CHD, cardiovascular mortality and cardiovascular risk factors consisting of diabetes, dyslipidemia, hypertension and central obesity compared to the general population [4–7]. This risk profile is probably due to higher rates of smoking, unhealthy lifestyle, physical inactivity, side effects to antipsychotic medicine [4, 6] and reduced compliance [8]. In European countries, cardiovascular mortality rates have declined over the last decades due to improved diagnostic methods, treatment options and lifestyle changes [9]. However, patients with schizophrenia receive less pharmacological treatment of cardiovascular risk factors and lower prescription rates of cardioprotective drugs following myocardial infarction compared to the general population [10, 11]. Late detection of risk factors [4] and higher prevalence of unrecognized myocardial infarction have been reported [12], which might be due to atypical presentation and lack of symptom recognition [13]. Thus, tools for early detection of CHD in patients with schizophrenia are needed to establish preventive strategies and reduce the risk of cardiovascular mortality. The amount of coronary artery calcium (CAC) has shown to be associated with CHD mortality independent of other cardiovascular risk factors in the general population [14–16]. The presence and extent of coronary artery calcium can be quantified by the use of noninvasive cardiac computed tomography (CCT) using the CAC score, which correlates with the actual amount of histologically detectable calcium in the coronary arteries detected post-mortem [17]. The CAC score has shown to improve cardiovascular risk assessment when added to traditional risk factors in the general population [15, 16, 18]. Established risk models for prediction of CHD may not be sufficient in the determination of cardiovascular risk in patients with schizophrenia [19], and measurement of CAC scores could possibly add prognostic information in the same way as in the general population. One other study showed no difference in CAC scores in a group of patients diagnosed with schizophrenia or bipolar disorder referred for CCT on clinical indication compared to other people with a registered CCT [20]. Traditional risk factors such as diabetes, dyslipidemia, smoking, sex and age have shown to be associated with the CAC scores in the general population [20, 21].

However, the potential role of CAC score measurement for early recognition of subclinical CHD has not yet been investigated in patients with schizophrenia screened without a clinical indication for conducting the diagnostic procedure.

In this study, we hypothesized that patients with schizophrenia have a higher coronary artery calcium score compared to the general population. Thus, the aim of this study was to investigate the coronary artery calcium score in patients with schizophrenia, who were not referred on clinical indication as compared to reference norms based on the general population. Moreover, we aimed to investigate if age, sex, diabetes, dyslipidemia and smoking were associated with the coronary artery calcium score.

## Methods

### Study design and subjects

In a clinical cross-sectional study, a total of 163 patients diagnosed with schizophrenia underwent CCT scan and measurement of the CAC score, which represents the total amount of CAC summed for the coronary arteries using densities and areas of calcified lesions. Based on the CAC score, the risk of future CHD is categorized into four categories: 0 = very low risk, 1–99 = mildly increased risk, 100–299 = moderately increased risk, 300–1000 = moderate to severely increased risk [22]. In addition, the score is commonly reported in relation to age- and gender matched percentiles, and values above the 75th percentile are of clinical interest in connection with increased risk of CHD [23–25].

First, the population was categorized into two groups (CAC score = 0 and CAC score > 0) representing the absence or presence of any CAC given the increased survival rates in patients with a CAC score of zero [26] and the general zero inflated distribution of the CAC score [27]. Second, the population was categorized in relation to the age- and gender matched CAC score norm percentiles used in Danish clinical practice [25].

Patients were included from outpatient clinics in the North Denmark Region and inclusion criteria were age ≥ 18 years, residency in the North Denmark Region and diagnosed with F20 (schizophrenia) or F25 (schizo-affective disorder) according to the International Classification of Diseases, Tenth Revision (ICD-10). Exclusion criteria were pregnancy, lactation, claustrophobia or inability to cooperate on the planned examinations. All participants provided written informed consent.

Data was collected at Aalborg University Hospital, Denmark between December 2015 and October 2019 as part of a clinical prospective cohort study conducted by the CardioSchizoStudyGroup [28].

### Assessment of coronary artery calcium

The CAC score was assessed from CCT scans with a 128-slice CT scanner (Siemens Healthcare GmbH, Germany). The examination was performed in supine position (tube voltage 120 kV, tube current 50 mAs, tube rotation time of 0.28 s) with a slice thickness of 3 mm and without intravenous contrast administration. Two observers (medical student TT and senior cardiologist SEJ) independently marked all calcified lesions with relation to the coronary arteries distinguishable from the aorta. The imaging analysis software SIEMENS Workstation syngo.via was used to calculate the CAC score as a sum of scores for all marked lesions with a minimum attenuation of 130 Hounsfield Units and a minimum area of two contiguous voxels according to the Agatston method [29]. This method is a well-established standardized CAC scoring method and is considered the gold standard for quantification of CAC [23, 30]. In the case of disagreement between the observers, images were reanalyzed to reach consensus.

### Baseline characteristics

The severity of schizophrenia was assessed by an experienced psychiatric nurse using the overall severity from the standardized Clinical Global Impression-Schizophrenia rating scale (CGI-SCH) [31]. The severity was given a rating of one to seven points categorizing the patients as either normal, not at all ill (one point), borderline mentally ill (two points), mildly ill (three points), moderately ill (four points), markedly ill (five points), severely ill (six points) or among the most extremely ill patients (seven points). Clozapine treatment indicating resistance to treatment was defined as present if current use of clozapine was reported. The duration of schizophrenia was defined as years since the patients were diagnosed with schizophrenia assessed by the first appearance of the diagnosis in the medical records. Blood samples were collected to determine serum concentration of glycated hemoglobin (HbA1c), total cholesterol, triglyceride, high-density lipoprotein (HDL) and low-density lipoprotein (LDL). Smoking was defined as present if the participant reported any current or previous smoking. Diabetes was defined as present if HbA1c ≥ 48 mmol/l, or if current antidiabetic medication was reported. Blood pressure was measured on each arm after ten minutes of rest and mean systolic blood pressure was calculated. Dyslipidemia was defined as present if current lipid lowering medication was reported, or if total cholesterol, triglyceride, HDL or LDL exceeded specific thresholds. The thresholds were determined from the estimated ten-year risk for fatal cardiovascular events and whether diabetes was present. The absolute risk was estimated in patients ≥40 years and the relative risk in patients < 40 years in accordance with the SCORE algorithm for low risk countries [32]. The estimations were based on age, total cholesterol, mean systolic blood pressure and smoking status. Hypertension was defined as present if mean systolic blood pressure was ≥140 mmHg, mean diastolic blood pressure was ≥90 mmHg or current antihypertensive medication was reported. Obesity was defined as present if body mass index (BMI) was ≥30 determined by height and weight.

### Statistical analysis

Baseline characteristics were analyzed according to type, calculating percentages and frequencies for categorical data and means and standard deviations (SD) for continuous data and for CGI-SCH points. The CAC score was reported as percentiles. Baseline characteristics in patients with a CAC score of zero were compared to baseline characteristics in patients with a CAC score above zero using chi-squared tests for categorical data and t-test for continuous data. The CAC scores were categorized into 0, 1–99, 100–299 and ≥ 300 and compared to age- and gender matched norm percentiles used in clinical practice in Denmark [25] graphically and by reporting the percentage of CAC scores below or equal to the 50th percentile, above the 75th percentile and above the 90th percentile. Previous studies found associations between age, male sex, diabetes, dyslipidemia, smoking and hypertension as explanatory variables and CAC score as outcome in the general population [20, 21]. We aimed to explore the association between these variables and the CAC score in patients with schizophrenia by regression analysis. The number of patients in the present study, especially the number of patients having a CAC score above zero (*n* = 49), led us to prioritize and limit the number of covariates included in the main analysis. Even though hypertension is relevant for the analysis, the forementioned prioritization led us to not include blood pressure. This was done, as we do believe that blood pressure is most likely to be influenced by external factors such as measurement in a clinical setting and most likely to fluctuate over time and. Thus, blood pressure measured in a clinical setting might not be good enough proxy for hypertension to warrant adding the extra covariate in the regression analysis. The CAC score was not normally distributed; however, CAC scores above zero were normally distributed after log-transformation determined using histogram, QQ-plot and Shapiro Wilk test for normality. Therefore, the regression analysis was performed in two steps, a method also applied in previous studies [21, 33]. First, a logistic regression model was utilized to assess the correlation between age, sex, diabetes, dyslipidemia and smoking as explanatory variables and a CAC score above zero as outcome. Second, a linear regression model was applied on log-transformed CAC scores above zero to assess the correlation between age, sex, diabetes, dyslipidemia and smoking as explanatory variables and the extent of coronary artery calcium as outcome measure. Both steps consisted of five univariate models with age, sex, diabetes, dyslipidemia and smoking respectively as explanatory variables and a CAC score above zero as outcome and one multivariate model with age, sex, diabetes, dyslipidemia and smoking as explanatory variables and CAC score as outcome. Results from the logistic regression were reported as odds ratios (OR) and coefficients from the linear regression were transformed to percentage change in CAC score to ease interpretation. A sensitivity analysis on treatment with lipid lowering medication, antidiabetic medication, serum concentration of HbA1c, triglyceride, HDL and LDL was carried out. Furthermore, a second posthoc sensitivity analysis was carried out including logistic and linear regression with hypertension, obesity, age, sex and smoking as explanatory variables and CAC score as outcome. Obesity was included in this analysis since one previous study [20] has shown association between BMI and the CAC score in patients with schizophrenia. Hypertension was included since previous studies have shown association with the CAC score in patients with schizophrenia and the general population [20, 34, 35] A *P*-value < 0.05 was considered statistically significant. All analyses were performed using STATA version 16 (StataCorp LP, College Station, Texas, USA).

## Results

This study included 163 patients (96 (58.9%) males). The mean age was 48.2 (SD ± 10.4) years, ranging from 24 to 75 years. The mean duration of schizophrenia was 19.7 (SD ± 8.2) years, ranging from 3 to 46 years. According to the CGI-SCH rating scale, one patient (0.6%) was categorized as normal, not at all ill, 23 (15%) as borderline mentally ill, 45 (29%) as mildly ill, 49 (32%) as moderately ill, 26 (17%) as markedly ill, 10 (6%) as severely ill and one (0.6%) as among the most extremely ill. A total of 41 (25.2%) patients received clozapine treatment. Further baseline characteristics are given in Table 1, and baseline characteristics stratified by group (CAC score = 0 and CAC score > 0) are given in Table 2. Less than 5% missing data was found on HbA1c, total cholesterol, triglyceride, HDL, LDL, smoking and lipid lowering medication due to withdrawal of consent, unwillingness or inability to participate in these examinations. This was considered missing at random, and imputation (mean for continuous variables and random for categorical variables) was carried out to deal with the missing data for regression modelling.

**Table 1:** Baseline characteristics

	Total
N (%)	163 (100)
Age, mean (SD)	48.2 (10.4)
Male sex, n (%)	96 (58.9)
Dyslipidemia, n (%)	109 (66.9)
Diabetes, n (%)	34 (20.9)
Smoking, n (%)	124 (76.1)
Hypertension, n (%)	62 (38.0)
Obesity, n (%)	63 (38.7)
LDL, unmeasurable, n (%)	12 (7.4)
mean (SD)	2.5 (0.9)
HDL, mean (SD)	1.3 (0.4)
Triglyceride, mean (SD)	2.3 (2.3)
CAC score	
25th percentile	0
50th percentile	0
75th percentile	6
90th percentile	114
Duration of schizophrenia	
n (%)	141 (100)
mean years (SD)	19.7 (8.2)
Severity of schizophrenia^a^	
n (%)	155 (100)
Normal, not at all ill (1 point)	1 (0.6)
Borderline mentally ill (2 points)	23 (15)
Mildly ill (3 points)	45 (29)
Moderately ill (4 points)	49 (32)
Markedly ill (5 points)	26 (17)
Severely ill (6 points)	10 (6)
Among the most extremely ill (7 points)	1 (0.6)
CGI-SCH score^a^, mean (SD)	3.7 (1.2)
Clozapine treatment, n (%)	41 (25.2)

^a^Based on the CGI-SCH rating scale for overall severity.

**Table 2:** Baseline characteristics comparison between groups CAC score = 0 and CAC score > 0

Variables	CAC score = 0	CAC score > 0	*P*-value
n (%)	114 (69.9)	49 (30.1)	
Age, mean (SD)	44.5 (9.1)	56.9 (7.7)	**< 0.001**
Male sex, n (%)	65 (57.0)	31 (63.3)	0.457
Dyslipidemia, n (%)	70 (63.6)	39 (79.6)	**0.045**
Diabetes, n (%)	20 (17.5)	14 (28.6)	0.112
Smoking, n (%)	80 (70.2)	44 (89.8)	**0.007**
Hypertension, n (%)	38 (33.3)	24 (48.8)	0.059
Obesity, n (%)	46 (40.4)	17 (34.7)	0.496
LDL, unmeasurable, n (%)	9 (8.4)	3 (6.1)	
LDL, measurable, n (%)	98 (91.6)	46 (93.9)	
mean (SD)	2.6 (0.9)	2.5 (0.9)	
HDL, mean (SD)	1.3 (0.4)	1.4 (0.5)	
Triglyceride, mean (SD)	2.2 (1.9)	2.5 (3.0)	
CAC score			
25th percentile		13	
50th percentile		35	
75th percentile		221	
90th percentile		783	
Severity of schizophrenia^a^			
CGI-SCH score^a^, mean (SD)	3.7 (1.1)	3.6 (1.3)	0.545
Duration of schizophrenia			
mean years (SD)	17. 8 (7.6)	24.1 (8.1)	< 0.001
Clozapine treatment, n (%)	33 (28.9)	8 (16.3)	0.089

^a^Based on the CGI-SCH rating scale for overall severity.

**Fig. 1:**
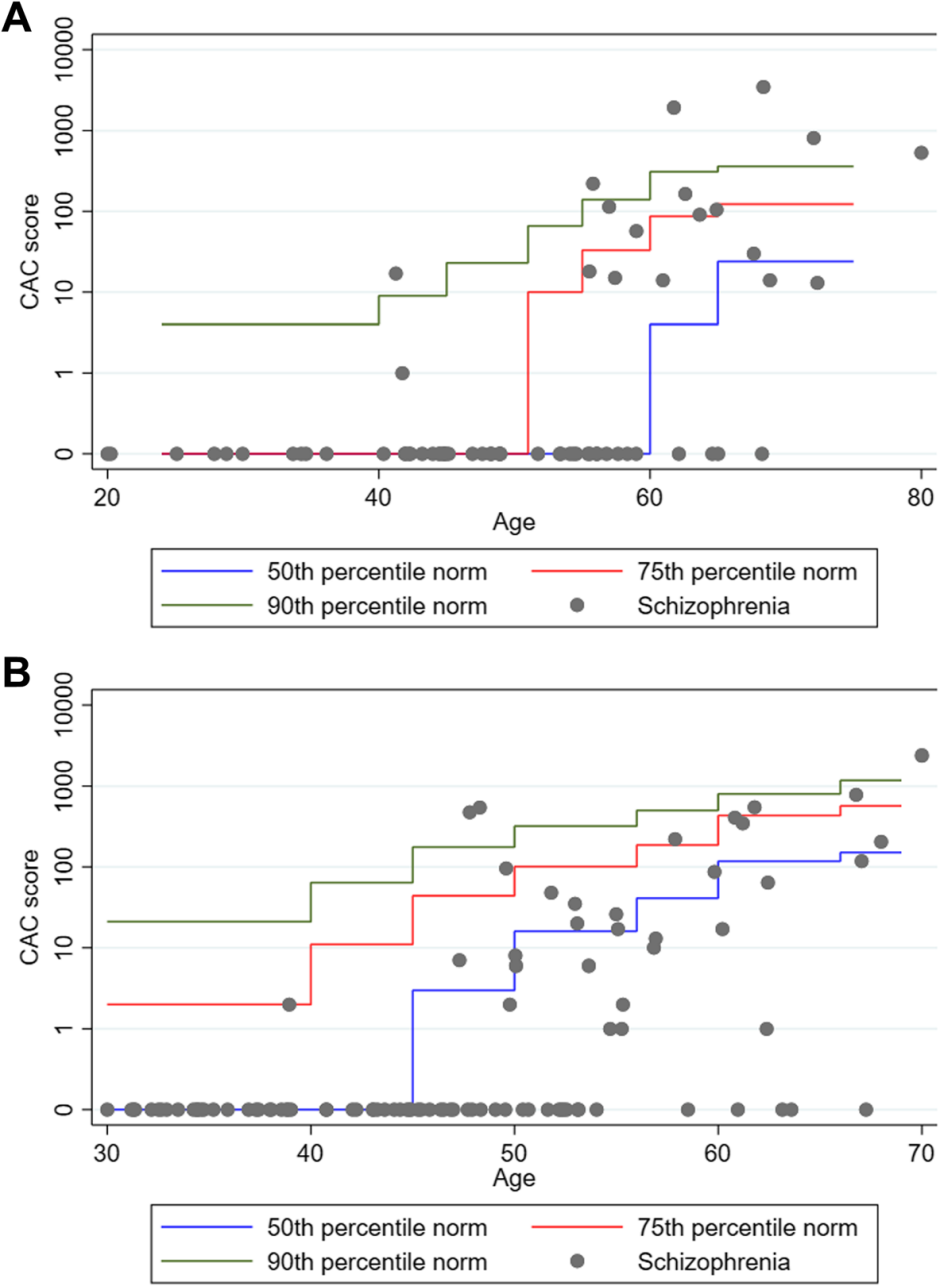
The CAC scores in patients with schizophrenia represented on a logarithmic scale in relation to age-specific norm 50th, 75th and 90th percentiles divided by sex. Zero was added on the otherwise logarithmic y-axis to show the large amount of patients having a CAC score of zero. **A** Females **B** Males

The CAC score ranged from zero to 3448 with a mean of 86.5 (SD ± 376), 25th percentile of zero, 50th percentile of zero, 75th percentile of six and 90th percentile of 114. A total of 114 (69.9%) patients had a CAC score of zero including 65 (39.9%) males and 49 (30.1%) females, 31 (19.0%) had a CAC score in the range 1–99, seven (4.3%) in the range 100–299 and 11 (6.8%) ≥ 300. A total of 127 (77.9%) patients including 76 (46.6%) males and 51 (31.3%) females had a CAC score below or equal to the age- and gender matched norm 50th percentile. All patients with a CAC score exactly equal to the age- and gender matched norm 50th percentile had a CAC score of zero. A total of 20 (12.3%) patients including seven (7.3%) males and 13 (19.4%) females had a CAC score above the age- and gender matched norm 75th percentile and one male patient (0.6%) had a CAC above zero and exactly equal to the 75th percentile. A total of nine (5.5%) patients including three (3.1%) males and six (9.0%) females had a CAC score above the norm 90th percentile. Fig. 1 illustrates the CAC scores in relation to age- and gender matched norm percentiles. Patients with a CAC score above zero were older (*P* < 0.001), had an increased duration of schizophrenia (*P* < 0.001) and higher rates of dyslipidemia (*P* < 0.05) and smoking (*P* < 0.01) than patients with a CAC score of zero.

### Regression analysis

In the univariate logistic regression models, age (OR 1.19; 95% CI 1.13–1.20; *P* < 0.001), dyslipidemia (OR 2.23; 95% CI 1.03–5.02; *P* < 0.05) and smoking (OR 3.74, 95% CI 1.36–10.3; *P* < 0.01) were significantly associated with a CAC score above zero. However, in the multivariate model, the association between dyslipidemia and a CAC score above zero was no longer significant (OR 1.84; 95% CI 0.66–5.14; *P* > 0.05). In the multivariate model, the association between age and a CAC score above zero remained significant (OR 1.20; 95% CI 1.13–1.20; *P* < 0.001), similar to the association between smoking and a CAC score above zero (OR 3.59; 95% CI 1.07–12.07; *P* < 0.05). In the model, male sex was also significantly associated with a CAC score above zero (OR 2.73; 95% CI 1.05–7.13; *P* < 0.05). An overview of the logistic regression analysis is given in Table 3.

**Table 3:** Logistic regression on the presence of CAC measured as CAC score

Variables	Univariate		Multivariate	
	OR (95% CI)	*P*-value	OR (95% CI)	*P*-value
Age	1.19 (1.12–1.26)	**< 0.001**	1.21 (1.13–1.29)	**< 0.001**
Male sex	1.33 (0.47–0.67)	0.415	3.15 (1.16–8.52)	**0.024**
Dyslipidemia	2.23 (1.01–4.94)	**0.048**	1.48 (0.52–4.23)	0.465
Diabetes	2.19 (0.98–4.90)	0.057	1.75 (0.60–5.16)	0.308
Smoking	3.94 (1.43–10.8)	**0.008**	3.95 (1.16–13.48)	**0.028**

The univariate linear regression of log-transformed CAC scores above zero (*n* = 49) showed that age and diabetes were significantly associated with the extent of coronary artery calcium measured as CAC score. In this model, a one-year increase in age was associated with a 15% increase in CAC score (95% CI 7–24%; *P* < 0.001), while diabetes was associated with a 577% increase in CAC score (95% CI 57–2020%; *P* < 0.01). Similar results were found in the linear regression multivariate model in which a one-year increase in age was associated with a 16% increase in CAC score, an association which remained statistically significant (95% CI 9–25%; *P* < 0.001). In the multivariate model, diabetes was associated with a 419% increase in CAC score which remained statistically significant (95% CI 68–1500%; *P* < 0.01). An overview of results from the linear regression models is given in Table 4. Sensitivity analyses (Table 5) showed no association between CAC score and LDL, triglyceride, HDL, statin treatment or HbA1c in multivariate logistic and linear regression. Antidiabetic medication was significantly associated with log-transformed CAC scores (*P* < 0.05), which is consistent with the association between diabetes and the extent of CAC found in the linear regression. The posthoc sensitivity analysis showed that hypertension and obesity were not associated with the CAC score (data not shown).

**Table 4:** Linear regression on coronary artery calcium measured as log-transformed CAC scores above zero (n = 49)

Variables	Univariate			Multivariate		
	Coeff (95% CI)	Percent increase in CAC score (95% CI)^a^	*P*-value	Coeff (95% CI)	Percent increase in CAC score (95% CI)^a^	*P*-value
Age	0.06 (0.03–0.09)	15% (7–24%)	**< 0.001**	0.07 (0.04–0.10)	16% (9–25%)	**< 0.001**
Male sex	−0.35 (−0.91–0.22)	−55% (−88–164%)	0.222	−0.05 (−0.53–0.44)	−11% (68–173%)	0.839
Dyslipidemia	0.08 (−0.60–0.76)	19% (−75–472%)	0.822	0.17 (− 0.40–0.73)	47% (−60–436%)	0.556
Diabetes	0.76 (0.20–1.33)	577% (57–2020%)	**0.009**	0.71 (0.23–1.20)	419% (68–1500%)	**0.005**
Smoking	0.05 (−0.55–1.25)	126% (−72–1695%)	0.434	0.55 (− 0.19–1.28)	250% (−35–1792%)	0.140

^a^Calculated as inversing log of the coefficients from the linear regression of log-transformed CAC scores and subtracting 1.

**Table 5:** Sensitivity analysis on CAC scores in multivariate logistic regression (*N* = 163) and log-transformed CAC scores above zero in linear (*n* = 49) regression

Variables	Logistic regression		Linear regression	
	OR (95% CI)	*P*-value	Coeff	*P*-value
LDL	1.21 (0.69–2.11)	0.505	−1.93 (−0.48–0.09)	0.178
Triglyceride	1.05 (0.87–1.27)	0.609	−0.01 (− 0.08–0.07)	0.849
HDL	0.93 (0.31–2.83)	0.905	−0.07 (− 0.60–0.46)	0.805
Statin treatment	1.38 (0.47–4.05)	0.561	−0.08 (− 0.58–0.41)	0.733
HbA1c	1.01 (0.97–1.05)	0.770	0.02 (−0.00–0.04)	0.101
Antidiabetic medication	1.69 (0.55–5.17)	0.357	0.66 (0.15–1.18)	0.013

## Discussion

In this study, the amount of coronary artery calcium in patients with schizophrenia follows age- and gender matched norms based on the general population with 77.9% below or equal to the norm 50th percentile, 12.3% above the norm 75th percentile and 5.5% above the norm 90th percentile. Variables independently associated with the presence of coronary artery calcium were male sex, age and smoking, and variables independently associated with the extent of coronary artery calcium were age and diabetes.

Similarly, previous studies found age, sex, smoking and diabetes to be associated with CAC score in the general population [20, 21, 36]. In a study by Kugathasan et al. [20] age, smoking, BMI and hypertension were found to be associated with the CAC score in a group of patients with schizophrenia or bipolar disorder all undergoing cardiac procedures on a clinical indication. However, hypertension and BMI applied in posthoc sensitivity analysis in the present study showed no association with the CAC score. One possible explanation for the contradictory results could be that patients included in this study were younger (mean of 48.2 years compared to 53.9 years) and thereby less likely to have developed CHD and increased risk of having a CAC score of zero. If more patients had a CAC score above zero, hypertension might have showed correlation with the CAC score as seen in previous studies [34]. The number of patients with a CAC score of zero was not reported in the study by Kugathasan et al., however the percentage prevalence of CAC scores in the range 0–99 (88.0%) were comparable to the present study (89.0%)Previous studies have shown both inverse correlations between BMI and CAC [37] and positive correlations between BMI and the presence of CAC [38] suggesting a complex relationship between body size and the CAC score possibly explaining conflicting findings. Lastly, the mechanism of developing CAC could differ between patients with schizophrenia, bipolar disorder (included in Kugathasan et al.) and the general population explaining differences in correlation between the CAC score, BMI and hypertension. However, Kugathasan et al. found no differences in CAC scores between patients diagnosed with schizophrenia or bipolar disorder and the background population [20]. Thus, the findings of CAC scores in relation to norm percentiles in this study are consistent with the existing literature on CAC scores in patients with schizophrenia.

We found a high prevalence (69.9%) of zero CAC score which has shown to be associated with lower all-cause mortality, lower rate of cardiovascular events and improved survival compared to a CAC score above zero [26, 39, 40]. In younger asymptomatic individuals aged ≤45 years, an adjusted all-cause mortality hazard ratio (HR) of 2.3 has been reported for individuals with even mild coronary artery calcium (CAC score 1–100) compared to individuals with no coronary artery calcium [41]. Hence, having CAC score of zero is interpreted as low risk of CHD and all-cause mortality in a clinical setting, which would be the case in 69.9% of the patients with schizophrenia included in this study. In contrast, several studies support higher prevalence of CHD and related mortality in patients with schizophrenia compared to the general population [1, 7, 42]. One explanation for the findings could be the high prevalence of smoking found in the present study (76.1%) given higher mortality rates in smokers compared to non-smokers in the general population even in the absence of coronary artery calcium and underestimation of CHD risk in smokers [43, 44]. The underestimation in smokers challenges the use of CAC score for estimating cardiovascular risk in patients with schizophrenia due to the increased prevalence of smoking [6]. Moreover, increased mortality rates were found in patients with schizophrenia or bipolar disorder irrespective of the CAC score with high rates of non-cardiovascular causes of death [20] suggesting minor importance of the CAC score in this population. The high prevalence of dyslipidemia (66.9%) found in this study further suggests competing risks of CHD with no association to the CAC score. Furthermore, an increased likelihood of not showing up to appointments at general practitioner and negative symptoms as part of the psychopathology such as social deprivation in patients with schizophrenia [45] could cause less control of blood lipids and less use of lipid-lowering treatment [11].

Another possible explanation for the high number of patients having a CAC score of zero could be a more frequent development of unstable CHD in patients with schizophrenia. Unstable CHD has shown to be primarily related to vulnerable atherosclerotic plaques that cannot be detected by CCT instead of dense calcified plaques [46]. The stabilizing role of coronary artery calcium in the atherosclerotic plaques might not be developed to the same extent in patients with schizophrenia due to a more rapid development of atherosclerosis. Moreover, the overall low amount of coronary calcium found in this study could possibly be explained by the relatively low mean age of the population (48.2 years) and patients as young as 24 years less likely to have developed detectable and calcified plaques. On the other hand, statin treatment has shown to facilitate plaque stabilization by progression of coronary artery calcium and thereby decrease the risk of future cardiovascular events [47], which could confound the results of this study in patients with high CAC scores since treatment with lipid lowering medication was included in the definition of dyslipidemia.

### Strengths and limitations

As the first study ever investigating the CAC score in a sample of patients with schizophrenia, who were not referred for CCT, thus eliminating referral bias, this study has several strengths. Data collection was conducted in a structured manner enabling definitions of diabetes, dyslipidemia and risk assessment according to gold standards. In addition, the population was characterized thoroughly, reporting the duration of schizophrenia, contact with outpatient clinics, treatment with clozapine and severity at the time of examination according to a standardized rating scale.

However, the findings of this study should be interpreted in the light of its limitations. Especially, results from the linear regression should be interpreted with caution due to the large amount of CAC scores of zero and thereby a small sample size and broad 95% CIs. The current data did not allow for further statistical test of the CAC score between the general population and patients with schizophrenia due to the distributional properties of the CAC score. Furthermore, age- and gender matched norm percentiles used in this study were based on an American population from Raggi et al. [25] described in studies by Valenti et al. [26] and Ronde et al [24]. and, therefore, the generalizability to Denmark might be limited. However, these percentiles were used in Danish clinical practice, and a recent meta-analysis pooling several CAC score nomograms suggested that median CAC scores for USA and Europe were similar [24]. Patients with known cardiovascular disease or symptoms were not excluded from this study and might not be comparable with the norm reference population. Data on smoking and medication was self-reported, i.e. response bias cannot be ruled out. Moreover, the data on smoking was limited, categorizing patients as previous or current smokers with no consideration of the amount or duration. In addition, selection bias may have occurred in terms of not including the most severe cases of schizophrenia due to inability to attend a long and demanding examination program. Patients with the most severe schizophrenia might have the poorest lifestyle and the highest CAC score and are possibly not included in this study. However, a mean duration of schizophrenia of 19.7 years with patients still having contact with outpatient clinics despite the long duration indicates severe disease. Results from the CGI-SCH rating scale showed an overall high severity of disease in the study population with a total of 86 (55%) patients categorized as either moderately, markedly, severely or among the most extremely ill. In addition, 41 (25.2%) patients received clozapine treatment indicating severe disease in terms of resistance to treatment and possibly higher severity than classified by the CGI-SCH rating scale. Thus, selection bias does not fully explain the finding of a high number of patients with a CAC score of zero, and it will be challenging to include patients with even more severe disease in future studies. Furthermore, patients with the highest CAC score might have died and therefore not been included in this study.

The Agatston scoring method used for CAC scoring in this study has been discussed in the literature, and some limitations have been recognized [30]. The total number of calcified plaques, the presence of coronary artery calcium in the proximal dominant coronary artery and increased number of coronary arteries with calcium have shown to be predictive of CHD-related events independently of the CAC score [48–50]. However, the Agatston scoring method is considered the gold standard for detection of coronary artery calcium [30].

In conclusion, the findings of this study support previous findings that predictors of CHD used in the general population might not be sufficient in patients with schizophrenia [19, 51]. Future studies should explore other measures of subclinical CHD to improve and initiate early prophylactic treatment and may include measures of peripheral atherosclerosis or cardiac autonomic neuropathy to improve early detection and intervention.

## Conclusions

Coronary artery calcium in patients with schizophrenia follows norm percentiles compared to the general population. Variables associated with the presence and extent of coronary artery calcium in the general population are similarly associated with coronary artery calcium in patients with schizophrenia. Further studies should address the progression of coronary artery calcium and other measures of subclinical cardiovascular disease in these patients, e.g. peripheral atherosclerosis or cardiac autonomic neuropathy to improve treatment and survival.

## Supplementary Information

**Additional file 1: Supplementary Fig. 1.** This chart is for use in people without cardiovascular disease, diabetes or very high levels of individual risk factors [1]. **Supplementary Fig. 2.** This chart is for use in younger people under the age of 40 since the absolute risk chart may underestimate cardiovascular risk in this population [2].

## Data Availability

The datasets generated and analyzed during the current study are not publicly available but are available from the corresponding author upon reasonable request.

## Abbreviations

CHD: Coronary heart disease
CAC: Coronary artery calcium
CCT: Cardiac computed tomography
ICD-10: International Classification of Diseases, Tenth Revision
CGI-SCH: Clinical Global Impression-Schizophrenia scale
HbA1c: Glycated hemoglobin
HDL: High-density lipoprotein
LDL: Low-density lipoprotein
