# Supplementary material for: Coronary artery calcium in patients with schizophrenia
Source: BMC Psychiatry. 2021 Aug 23;21:422. doi: 10.1186/s12888-021-03412-x (PMC8381587; doi:10.1186/s12888-021-03412-x)
Supplement: Supplementary file 1 — Additional file 1: Supplementary Fig. 1. This chart is for use in people without cardiovascular disease, diabetes or very high levels of individual risk factors [1]. Supplementary Fig. 2. This chart is for use in younger people under the age of 40 since the absolute risk chart may underestimate cardiovascular risk in this population [2]. [file 12888_2021_3412_MOESM1_ESM.docx]

**The SCORE algorithm**

The SCORE (Systematic Coronary Risk Estimation) charts are used to estimate 10-year risk of fatal cardiovascular disease (CVD). We calculated SCOREs for all patients using mean systolic blood pressure, total serum cholesterol, age, sex and smoking status. The absolute risk (Supplementary figure 1) was calculated for patients above 40 years and the relative risk (Supplementary figure 2) was calculated for younger patients in accordance with guidelines [1, 2].


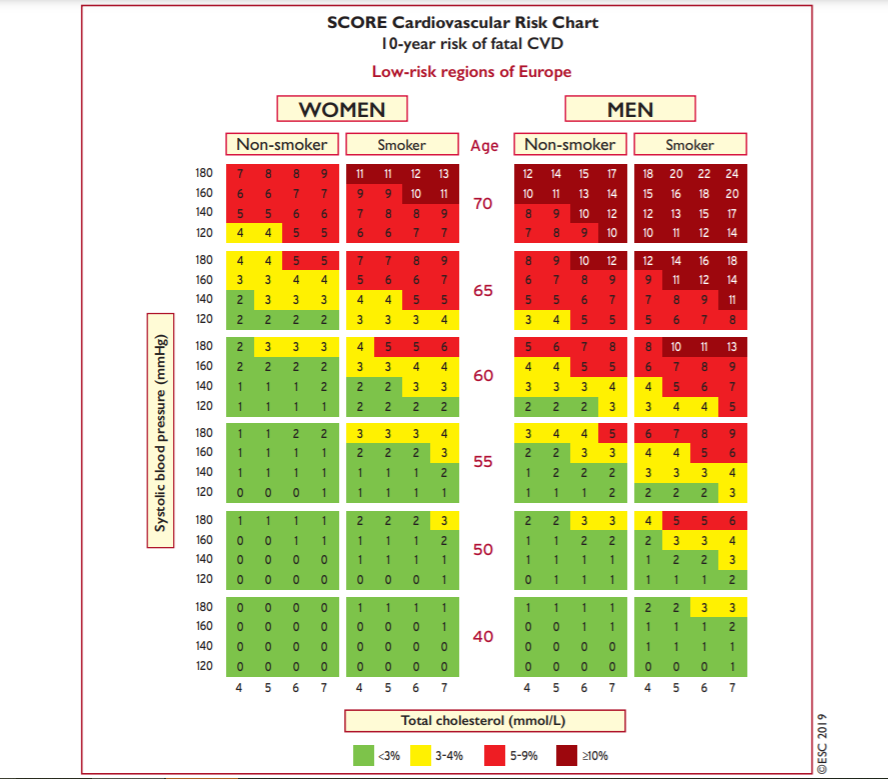


**Supplementary figure 1.** This chart is for use in people without cardiovascular disease, diabetes or very high levels of individual risk factors[1].


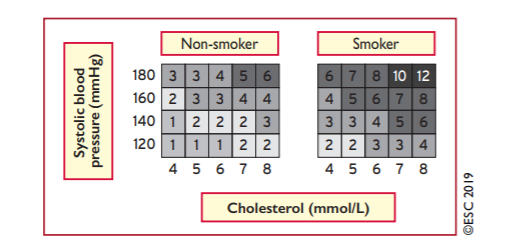


**Supplementary figure 2.** This chart is for use in younger people under the age of 40 since the absolute risk chart may underestimate cardiovascular risk in this population [2]

**Thresholds for dyslipidemia**

Dyslipidemia was considered present if guidelines would recommend statin treatment based on the SCORE, whether diabetes was present and thresholds for serum levels of LDL, total cholesterol, HDL or triglyceride. The following definitions are based on published guidelines[1].

- For individuals at low risk (SCORE<1), dyslipidemia was considered present if LDL was ≥3.0 mmol/L
- For individuals at moderate risk (SCORE 1-4), dyslipidemia was considered present if LDL was ≥2.6 mmol/L
- For individuals at high risk (SCORE 5-10 or mean systolic blood pressure ≥180), dyslipidemia was considered present if LDL was ≥1.8 mmol/L
- For individuals at very high risk (SCORE≥10), dyslipidemia was considered present
- Individuals with diabetes who reported smoking, this was considered an additional risk factor and the person was placed in the “high risk” category and dyslipidemia was considered present if LDL was ≥ 1.8
- Individuals with diabetes who reported no smoking was placed in the “moderate risk” category and dyslipidemia was considered present if LDL was ≥ 2.6
- For individuals with one markedly elevated single risk factor including serum total cholesterol ≥8 or serum LDL≥5, dyslipidemia was considered present
- Dyslipidemia was considered present if serum-triglyceride was > 1.7
- For males at low risk (SCORE<1), dyslipidemia was considered present if serum HDL was < 1.2
- For females at low risk (SCORE<1), dyslipidemia was considered present if serum HDL was < 1.0

**References**

[1] Mach F, Baigent C, Catapano AL, et al. 2019 ESC/EAS Guidelines for the management of dyslipidaemias: lipid modification to reduce cardiovascular risk. *Eur Heart J* 2019; 48: 121–127. https://doi.org/10.1093/eurheartj/ehz455

[2] Mach F, Baigent C, Catapano AL, et al. 2019 ESC/EAS Guidelines for the management of dyslipidaemias: lipid modification to reduce cardiovascular risk: supplementary data. *Eur Heart J* 2019; 00: 5. https://doi.org/10.1093/eurheartj/ehz455
